# The Association Between Psoriasis and Risk of Cardiovascular Disease: A Mendelian Randomization Analysis

**DOI:** 10.3389/fimmu.2022.918224

**Published:** 2022-06-29

**Authors:** Ning Gao, Minjian Kong, Xuebiao Li, Xian Zhu, Dongdong Wei, Ming Ni, Yifan Wang, Ze Hong, Aiqiang Dong

**Affiliations:** Department of Cardiovascular Surgery, The Second Affiliated Hospital of Zhejiang University School of Medicine, Hangzhou, China

**Keywords:** psoriasis, Mendelian randomization (MR), the causal link, cardiovascular disease, genome-wide association study (GWAS)

## Abstract

**Background:**

A large number of observational studies showed that patients with psoriasis have a higher risk of cardiovascular disease (CVD), but most studies did not fully adjust for confounding factors, so it is not clear whether the risk of CVD is directly attributed to psoriasis. We used Mendelian randomization (MR) to evaluate the potential causal relationship between psoriasis and CVD.

**Methods:**

We used genetic instruments from the genome-wide association study (GWAS) of European descent for psoriasis to investigate its relationship with CVD. Inverse variance-weighted (IVW) MR analyses were used for the primary analysis. In addition, a variety of other methods were used to replicate the analysis.

**Results:**

The fixed-effects IVW method indicated that genetic susceptibility to psoriasis was associated with a higher risk of heart failure (HF) [odds ratio (OR) = 1.04; 95% CI, 1.01–1.06, P = 2.72E-03], atrial fibrillation (AF) (OR = 1.04; 95% CI, 1.02–1.07, P = 3.27E-04), myocardial infarction (MI) (OR = 1.07; 95% CI, 1.01–1.12, P = 0.01), valvular heart disease (VHD) (OR = 1.001; 95% CI, 1.000–1.002, P = 1.85E-03), and large artery stroke (LAS) (OR = 1.11; 95% CI, 1.05–1.18, P = 5.37E-04) but not with the other two subtypes of ischemic stroke (IS) [cardioembolic stroke (CES) (OR = 1.03; 95% CI, 0.98–1.07, P = 0.27) and small vessel stroke (SVS) (OR = 1.00; 95% CI, 0.95–1.07), P = 0.88)]. Sensitivity analysis found weak evidence of horizontal diversity and heterogeneity to ensure the stability of the results.

**Conclusion:**

Our study provided evidence for a potential causal link between psoriasis and CVD. These findings partly suggest that early monitoring of cardiovascular risk in patients with psoriasis is intentional.

## Introduction

Cardiovascular disease (CVD) was defined as a group of cardiac and vascular diseases, including ischemic stroke (IS), atrial fibrillation (AF), heart failure (HF), myocardial infarction (MI), valvular heart disease (VHD), and so on (1). In 2020, about 19 million deaths were attributed to CVD globally. This estimate represented an 18.7% increase in the number of people dying from CVD in the decade leading up to 2020 (1–3). There were differences in cardiovascular mortality among different regions: the mortality rate of CVD in Eastern Europe and Central Asia was the highest, while that in North America and Western Europe was relatively low; meanwhile, the mortality rate of men (median 551/100,000) was higher than that of women (median 441/100,000) (2). The prevalence of CVD varied from population to population: American Indians or Alaskan natives had the highest prevalence (14.6%), while Asians had the lowest (7.7%). Currently, CVD has become one of the leading causes of death and disability in the world, accounting for 37% of deaths from non-communicable diseases under the age of 70, causing a huge economic burden to society (4, 5). CVD was often the result of a combination of multiple etiologies (6). The occurrence and progression of CVD might be driven by the interaction of genetic factors, environmental induction, and immune disorders (7).

Psoriasis was a chronic inflammatory skin disease characterized by the appearance of well-demarcated red scaly patches on the skin (8). More than 60 million people worldwide were affected by psoriasis, and the prevalence varied across regions, with the highest in Oceania, Western Europe, Central Europe, and North America and lower in East Asia (9). The epidemiological difference of psoriasis was mainly due to the genetic background of the subjects (10). More than 80 psoriasis susceptibility loci have been identified by the latest genome-wide association studies (GWASs), and these data explained about 30% of all heritability (11).

In recent years, the association between psoriasis and CVD has been paid more and more attention (12). Compared with the general population, CVD was more easily observed in patients with psoriasis. A prospective cohort study of 130,000 patients with psoriasis and 500,000 controls reported an overall 50% increased risk of MI in patients with psoriasis [odds ratio (OR) = 1.50] (13). The risk of HF in psoriatic patients was 63% higher than that in the control group (OR = 1.63) (14). Similarly, a large cohort study noted higher rates of IS and AF in patients with psoriasis compared with the general population (15). A similar finding was reported on the risk of VHD in patients with psoriasis (16). However, some studies refuted the association between psoriasis and CVD risk. In a case–control study with an 11-year follow-up, there was no increased risk of CVD events (MI, IS, and HF) in patients with predominantly mild psoriasis (17). A cohort study of 48,523 patients with psoriasis and 208,187 controls indicated that psoriasis was not associated with CVD risk after adjustment for known CVD risk factors (18).

Notably, whether CVD risk is directly attributable to psoriasis remains to be determined. This is because traditional risk factors for cardiovascular disease (including smoking, obesity, dyslipidemia, and stress) are often risk factors for psoriasis as well (19). These confounding factors were not adequately adjusted for by these studies, which could lead to spurious associations.

Confirmation of a causal association is as challenging as the reverse causation and confounding between psoriasis and the risk of CVD. As a new epidemiological research method, Mendelian randomization (MR) could explain observational bias, which used genetic variation as instrumental variables (IVs) to assess the causal effects of exposure factors on outcomes (20, 21). In addition, because of the unique advantage of tool variables, MR relies on the random assignment of genes during meiosis, resulting in a random distribution of genetic variations in a population (20). MR analysis could largely overcome the interference of traditional confounding factors (22) and accords with the normal causal order (23, 24). Evolving GWASs have also provided robust and reliable IVs for MR studies. In the present study, we conducted a two-sample MR study to explore whether genetic evidence of psoriasis was significantly associated with CVD risks.

## Methods

### Data Resources and Study Design

Summary statistics data for psoriasis were derived from FinnGen (https://r5.finngen.fi/), including 4,510 cases and 212,242 controls. For the outcome dataset, GWAS data for HF were derived from HERMES Alliance (25), including 47,309 cases of European origin and 930,014 controls. Single-nucleotide polymorphisms (SNPs) for AF were derived from a large meta-analysis of GWAS (26), including 65,446 cases and 522,744 controls. Summary-level data for MI were derived from CARDIoGRAMplusC4D that included 60,801 cases and 123,504 controls (27). Summary statistics for VHD were derived from UK Biobank (Neale lab) (http://www.nealelab.is/uk-biobank), including 1,606 cases and 359,588 controls. The summary dataset for IS was from the MEGASTROKE consortium, including three subtypes: large artery stroke (LAS) (4,373 cases), cardioembolic stroke (CES) (9,006 cases), and small vessel stroke (SVS) (5,386 cases) (28). An overview of the demographics involved in this study is shown in **Table 1**, and **Supplementary Table S1** presents a description of the GWAS included in this study.

**Table 1:** Data sources and instrumental variable strength assessment.

Traits	Data sources	Sample size (cases/controls)	Ancestry	Gender	*F*(Total)
**Exposure**					
Psoriasis	FinnGen	4,510/212,242	European	Men and women	
**Outcomes**					
Heart failure	HERMES	47,309/930,014	European	Men and women	14.71
Atrial fibrillation	AFGen	65,446/522,744	European	Men and women	13.65
Myocardial infarction	CARDIoGRAMplusC4D	60,801/123,504	77% European	Men and women	12.25
Valvular heart disease	Neale lab (UK Biobank)	1,606/359,588	European	Men and women	14.11
Large artery stroke	MEGASTROKE	4,373/406,111	European	Men and women	19.17
Cardioembolic stroke	MEGASTROKE	7,193/406,111	European	Men and women	19.17
Small vessel stroke	MEGASTROKE	5,386/406,111	European	Men and women	19.17

CARDIoGRAMplusC4D, Coronary Artery Disease Genome-wide Replication and Meta-analysis plus The Coronary Artery Disease Genetics;

HERMES, Heart Failure Molecular Epidemiology for Therapeutic Targets; AFGen, Atrial Fibrillation Genetics; F, R^2^(N-K-1)/[K(1-R^2^)], R^2^, 2 × (1-EAF) × EAF × (β/SD)^2^, SD=SE × N^1/2^, where EAF is the effect allele frequency, β is the estimated effect on psoriasis, N is the sample size of the GWAS, and SE is the standard error of the estimated effect.

This two-sample MR study was conducted to evaluate the causal association between genetic susceptibility to psoriasis and the CVD risks. SNP was used as our IV (24). The whole process satisfies the three main hypotheses of classical MR analysis (29): 1) IVs directly affect the exposure; 2) IVs are not associated with confounders; 3) IVs influence the risk of the outcomes directly through the exposure, not through other pathways. All involved GWASs obtained ethical approval and informed consent. This study was reported in accordance with the latest Strengthening the Reporting of Observational Studies in Epidemiology Using Mendelian Randomization (STROBE-MR) guideline (30).

### Selection of Instrumental Variables

All SNPs significantly associated with psoriasis (P < 5 × 10^−8^) were selected as IVs. The corresponding linkage disequilibrium was tested to confirm that there were SNPs in a linkage disequilibrium state and that the SNPs were independent by pruning SNPs within a 10,000-kb window with an r^2^ < 0.001 threshold. To remove potential pleiotropic effects, we retrieved the secondary phenotype of each SNP in PhenoScanner V2 (31) and the GWAS Catalog. SNPs corresponding to the phenotypes associated with the results were excluded, and the remaining SNPs were used for further analysis.

Variance (*R^2^*) and *F*-statistic were used to assess the strength of IVs to avoid weak tool bias (32, 33). We adopted the latest and most stringent calculation method. *F* = *R* ^2^(*N*-*K*-1)/[*K*(1-*R* ^2^)]. In this equation, *R^2^* refers to the cumulative explained variance of the selected SNP during exposure, *K* is the number of SNPs for the final analysis, and *N* is the number of samples of the selected GWAS. *F*-statistic greater than 10 was considered to be sufficiently strong for the correlation between IVs and exposure that the results of the MR analysis could avoid being affected by weak tool bias.

### Statistical Analyses

We harmonized the summary SNP-psoriasis and SNP-CVD statistics to ensure effect size alignment and to prohibit strand mismatch. In MR analysis, the inverse variance-weighted (IVW) method of different models was used as the main analysis method according to heterogeneity (24). At the same time, median weighting (34), MR-Egger (35), maximum-likelihood (36), MR-robust adjusted profile score (MR-RAPS) (37), and MR-pleiotropy residual sum and outlier (MR-PRESSO) (38) were also used to evaluate the robust effects. Each method made different assumptions about the effectiveness of the IVs. The median weighting method can draw a reliable conclusion with at least 50% of the weight of the analysis coming from valid IVs (34). Although the statistical ability of the MR-Egger method is low, it provides an estimate after correcting the multiple effects (35). MR-RAPS corrects horizontal multiplicity by using robust adjusted contour scores, which reduces the deviation caused by horizontal multiplicity (37). The MR-PRESSO method can verify the results in the IVW model, correct the influence of outliers, and generate reliable heterogeneous causal estimates (38). In a word, we used all of these methods to study causality comprehensively.

### Sensitivity Analyses

Various methods were introduced into this study for sensitivity analysis. Firstly, Cochran’s Q test was used to assess heterogeneity between estimates of individual genetic variants. If the P value was <0.05, the final results of MR referred to the random-effects model of IVW; otherwise, the fixed-effects model (39). Secondly, we used the MR-Egger intercept method to test the horizontal pleiotropy of IVs (35). Thirdly, the leave-one-out sensitivity test was performed to check whether the results were caused by any single SNP. Fourth, funnel plots and forest plots were generated to directly detect the existence of pleiotropy.

All statistical analyses were performed using the “TwoSampleMR (0.5.6),” “MR-PRESSO (1.0),” and “mr.raps” packages in R, Version 4.1.2. All P values were two-sided, and P < 0.05 was deemed as suggestive of significance.

## Results

### Characteristics of the Selected Single-Nucleotide Polymorphisms

We extracted IVs significantly related to psoriasis from the GWAS study (P < 5 × 10^−8^) and removed linkage disequilibrium (LD) (r^2^ < 0.001, 10,000 kb). Subsequently, in the PhenoScanner database and GWAS catalog, we did not find any IVs for psoriasis to be associated with potential confounders. Meanwhile, palindromic SNPs (SNPs whose alleles consist of a base and its complementary base) were excluded. To maintain consistency of SNPs used as IVs across analyses, we only used variants that were available for all examined traits and did not replace missing variants with proxies. Finally, the screened SNPs were included in further analysis (**Supplementary Tables S1–S7**). No evidence of weak tool bias was found in the IV strength test (*F*-statistic >10) (**Table 1**).

### Causal Effects of Genetic Predisposition to Psoriasis With Risk of Cardiovascular Disease

**Figure 1:**
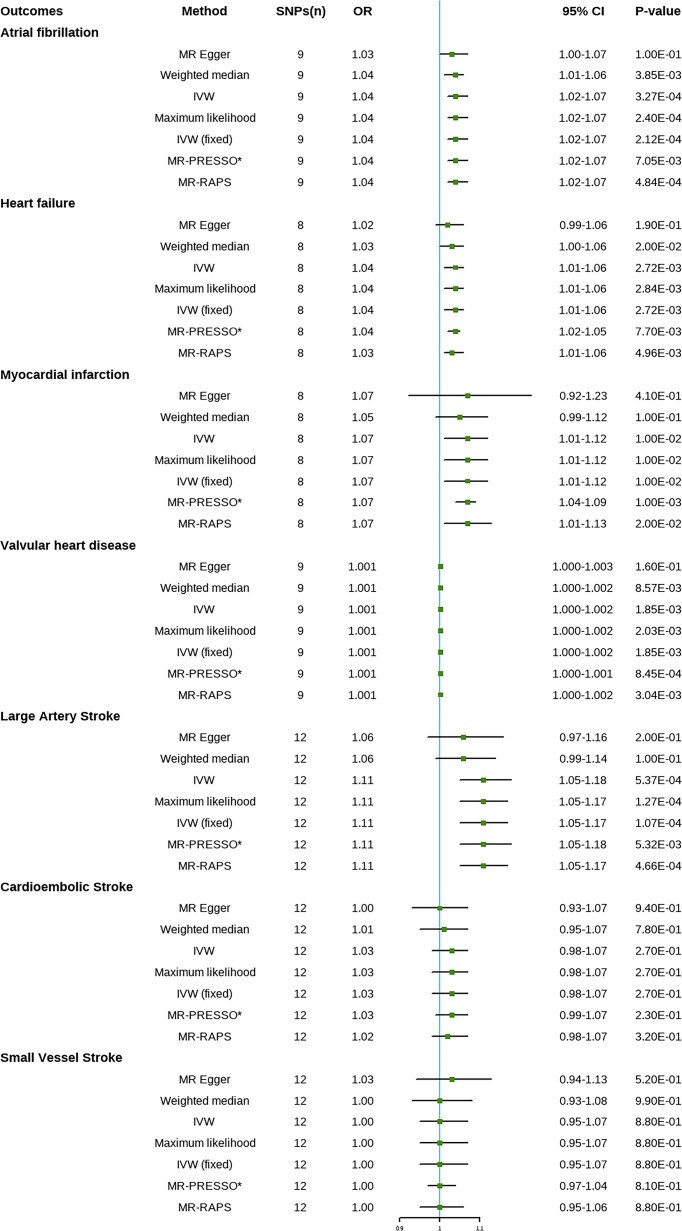
Mendelian randomization estimates of psoriasis on the risk for cardiovascular disease (CVD). SNPs, single-nucleotide polymorphisms; OR, odds ratio; CI, confidence interval; IVW, inverse variance-weighted; IVW (fixed), fixed-effects inverse variance-weighted; MR-RAPS, MR-robust adjusted profile score; MR-PRESSO, MR-pleiotropy residual sum and outlier. *No outlier was detected.

The statistical results of MR are presented in **Figure 1**. The fixed-effects IVW method indicated that genetic susceptibility to psoriasis was associated with a higher risk of HF, AF, MI, VHD, and LAS but not with the other two subtypes of IS (CES and SVS).

Compared with the control group, the prevalence of HF in psoriasis patients was 1.04 times (OR = 1.04; 95% CI, 1.01–1.06, P = 2.72E-03), the prevalence of AF was 1.04 times (OR = 1.04; 95% CI, 1.02–1.07, P = 3.27E-04), the prevalence of MI was 1.07 times (OR = 1.07; 95% CI, 1.01–1.12, P = 0.01), the prevalence of VHD was 1.001 times (OR = 1.001; 95% CI, 1.000–1.002, P = 1.85E-03), and the prevalence of LAS was 1.11 times (OR = 1.11; 95% CI, 1.05–1.18, P = 5.37E-04). There was no significant difference in the prevalence of CES (OR = 1.03; 95% CI, 0.98–1.07, P = 0.27) and SVS (OR = 1.00; 95% CI, 0.95–1.07, P = 0.88) between psoriasis patients and the controls. Weighted median, maximum likelihood, MR-PRESSO, and MR-RAPS analysis showed similar results to IVW method. No outliers were identified by MR-PRESSO method, indicating that the results were reliable. Risk calculations were performed based on the LogOR of psoriasis, which may partly explain the low OR values.

### Sensitivity Analyses of Mendelian Randomization

Sensitivity analyses indicated that there was no underlying heterogeneity and horizontal pleiotropy in the results (**Table 2**) and that the causal relationship between psoriasis and CVD risk was not driven by a single SNP (**Supplementary Figure S1**). The P values of the Cochran’s Q statistic were all greater than 0.05, indicating that there was no heterogeneity among IVs. Therefore, the fixed-effects IVW method was considered as the primary analysis method. Meanwhile, the MR-Egger regression intercept test showed that there was no evidence of horizontal pleiotropy in the IVs of psoriasis in any type of CVD. Forest plots and funnel plots were shown in **Supplementary Figures S2, S3**.

**Table 2:** Pleiotropy and heterogeneity test of the psoriasis genetic IVs from CVD GWAS.

Outcomes	Pleiotropy test			Heterogeneity test					
	MR-Egger			MR-Egger			Inverse variance-weighted		
	Intercept	SE	P	Q	Q_df	Q_P	Q	Q_df	Q_P
Heart failure	0.005	0.006	0.41	3.80	6	0.70	4.60	7	0.71
Atrial fibrillation	0.004	0.006	0.46	7.82	7	0.35	8.50	8	0.39
Myocardial infarction	0.001	0.016	0.99	1.58	6	0.95	1.58	7	0.98
Valvular heart disease	-8.46E-05	1.82E-04	0.66	2.68	7	0.91	2.89	8	0.94
Large artery stroke	0.02	0.02	0.22	11.79	10	0.30	13.77	11	0.25
Cardioembolic stroke	0.01	0.01	0.31	6.11	9	0.73	7.28	10	0.70
Small vessel stroke	-0.01	0.01	0.47	5.34	10	0.87	5.90	11	0.88

df, degree of freedom; MR, Mendelian randomization; Q, heterogeneity statistic Q.

## Discussion

This MR study demonstrated that one unit increase in log odds of psoriasis was associated with higher risks of HF, AF, MI, VHD, and LAS; furthermore, there is no evidence to support an association between psoriasis and the risk of SVS and CES.

The relationship between psoriasis and CVD was first described in the 1970s (40), followed by a series of observational studies to explore the possible relationship between the two. Cross-sectional studies showed that patients with psoriasis of any age had a higher risk of MI (41). Other prospective cohort studies also reached the same conclusion, and the incidence of MI increased with the severity of psoriasis (13, 42–44). The association between psoriasis and HF was not as clear as the rest of CVD. Compared with the general population, patients with psoriasis tended to be more likely to have HF, with hazard ratios of 1.22 and 1.53 for mild and severe psoriasis, respectively (14, 45). A UK population-based cohort study identified psoriasis as an independent risk factor for stroke (46). Likewise, this study also showed that patients with severe psoriasis (HR = 1.43) had a higher risk of stroke than those with mild psoriasis (HR = 1.06) (46). A large cohort study of 39,558 cases and 4,478,926 controls noted a higher incidence of AF in patients with psoriasis (15). A meta-analysis of 33 observational studies showed that patients with psoriasis had a higher risk of MI, HF, and IS (47), which is consistent with our findings. Another meta-analysis of 13 studies showed an increase in overall cardiovascular risk in patients with psoriasis (48). On the other hand, there are also studies denying the relationship between psoriasis and CVD. The risk of CVD was not increased in patients with psoriasis after adjustment for known risk factors (17, 18, 49).

The exact mechanism by which psoriasis increases the risk of CVD is unclear. Dyslipidemia in patients with psoriasis may contribute to the increased risk of CVD. Studies have shown that patients with psoriasis have elevated low-density lipoprotein, very low-density lipoprotein, and lipoprotein (a), accompanied by a decrease in high-density lipoprotein (50). Abnormal platelet activation was another possible cause of the high incidence of CVD in patients with psoriasis (51). Increased mean platelet volume was found in patients with psoriasis, which was associated with acute MI (52). Immune-mediated systemic inflammation may affect angiogenesis, lipid metabolism, and cardiac metabolism (53), while pro-inflammatory factors tumor necrosis factor-α (TNF-α) and interferon-γ (IFN-γ) may be a bridge between them (54). An clinical randomized trial (RCT) indicates that psoriatic patients treated with TNF inhibitors have a significantly lower risk of MI (HR = 0.50) (55). The systemic inflammatory response of psoriasis needs to be fully explored, especially whether biological agents have an exact effect on the prevention of CVD.

Patients with psoriasis tend to have more risk factors for CVD (smoking, abdominal obesity, diabetes, etc.). In a clinical trial, 59% of participants had at least 2 traditional CVD risk factors, while 29% had 3 or more (56). A recent MR analysis showed that increased body mass index was significantly associated with a higher risk of psoriasis (57). Another MR study identified a causal relationship between psoriasis and type 2 diabetes(P = 1.6 × 10^−4^, OR = 1.01) (58), which is one of the risk factors for CVD. As the vast majority of studies failed to fully adjust the confounding factors, the potential causal relationship between psoriasis and CVD has not been determined. However, it was necessary to understand the relationship between them, which could provide evidence for doctors to decide whether patients with psoriasis should be screened for CVD.

There are some strengths in our study. First, our MR analysis explored the causal relationship between psoriasis and a series of CVDs for the first time, and the results were unlikely to be influenced by confounders and reverse causal associations. Second, we used the latest large GWAS dataset, and the exposed data did not overlap with the outcome, which improved the reliability of the results. Third, the IVs of each group were evaluated to ensure that there was no tool bias. Fourth, multiple analytical methods were used to perform repeated analyses with consistent results. Furthermore, sensitivity analysis proved our results to be reliable.

However, our study also has some limitations. Firstly, although we used multiple steps to test for pleiotropy, the effect of potential pleiotropy could not be completely ruled out, resulting in an inaccurate assessment of the three hypotheses. Fortunately, multiple methods yielded consistent results and sensitivity analyses found weak evidence for horizontal pleiotropy, minimizing the possibility of pleiotropy bias. Secondly, the vast majority of participants in this MR analysis were from Europe, making it more difficult to explain the causal relationship between psoriasis and CVD in other populations. Thirdly, the OR value is relatively low, which should be interpreted carefully. We look forward to more in-depth research on the potential relationship between the two in the future.

## Conclusion

In summary, our study provides evidence for a potential causal association between psoriasis and CVD. Combined with evidence from observational studies, early cardiovascular risk assessment and prevention in patients with psoriasis are of interest, which facilitate the introduction of individual-specific treatments as soon as possible. Due to the low OR value, caution is required when generalizing the results.

## Data Availability Statement

The original contributions presented in the study are included in the article/**Supplementary Material**. Further inquiries can be directed to the corresponding author.

## Author Contributions

NG, MK, and AD designed the study and drafted the article. DW, MN, and MK conducted data acquisition. NG, MK, XL, DW, MN, ZH, XZ, YW, and AD performed data analysis and article revision. All authors read and approved the final article.

## Funding

This research was funded by Zhejiang Health Major Science and Technology Program, National Health Commission Scientific Research Fund (WKJ-ZJ-2121), and the National Natural Science Foundation of China (81800210).

## Conflict of Interest

The authors declare that the research was conducted in the absence of any commercial or financial relationships that could be construed as a potential conflict of interest.

## Publisher’s Note

All claims expressed in this article are solely those of the authors and do not necessarily represent those of their affiliated organizations, or those of the publisher, the editors and the reviewers. Any product that may be evaluated in this article, or claim that may be made by its manufacturer, is not guaranteed or endorsed by the publisher.
