# Supplementary material for: The Association Between Psoriasis and Risk of Cardiovascular Disease: A Mendelian Randomization Analysis
Source: Front Immunol. 2022 Jun 29;13:918224. doi: 10.3389/fimmu.2022.918224 (PMC9278135; doi:10.3389/fimmu.2022.918224)
Supplement: Supplementary file 1 [file DataSheet_1.docx]

Supplementary Material

## SupplementaryTable

Supplementary Table 1 Characteristics of the genome wide association studies used in this study.

| **Exposure** | **Data source**  **(or PMID)** | **Inclusion Criteria for SNP** | **Imputation Quality Control**  **(r2/ INFO score)** | **Case Definition** | **Unit** | **Covariate adjustments** | **Imputation platfrom** |
| --- | --- | --- | --- | --- | --- | --- | --- |
| Psoriasis | FinnGen | MAF >1% | info score > 0.6 | International Classification of Diseases (ICD10) L40.0 | logodds | sex, age, first 10 principal components, genotyping batch, and genetic relatedness | The 1000G Phase1 reference panels |
| **Outcomes** | **Data source (or PMID)** | **Inclusion Criteria for SNP** | **Imputation Quality Control**  **(r2/ INFO score)** | **Case Definition** | **Unit** | **Covariate adjustments** | **Imputation platfrom** |
| Heart failure | 31919418 | Study specific | Study specific INFO > 0.5 or 0.8 | Clinical Diagnosis of Heart Failure of any aetiology with no inclusion criteria based on LV ejection fraction | logodds | Sex, age (except for single-sex studies) and PCs for appropriate individual studies | 1KGP phase 1 or 3, Hapmap 2 NCBI build 36, Haplotype Reference Consortium, the Estonian WholeGenome Sequence reference, a reference sample based on 15,220 WGS of Icelandic individuals |
| Valvular heart disease | UK Biobank  (Neale lab) | MAF >1% | INFO >0.3 for MAF >3% INFO >0.6 for MAF 13% INFO >0.8 for MAF 0.5-1% INFO >0.9 for MAF 0.1-0.5% | Defined as Venous thromboembolism based on clinical and imaging criteria | logodds | first 20 principal components, sex, age, age^2^, interaction between sex and age, and interaction between sex and age squared | UK10K haplotype and HRC reference panels |
| Atrial fibrillation | 30061737 | Study  specific | r2 > 0.3 | DiscovEGR participants with at least one electronic health record problem list entry or at least two diagnosis code entries for two separate clinical encounters on separate calendar days for ICD-10 I48: atrial fibrillation and flutter. | logodds | Sex , age, age^2^, first four PCs of ancestry | Haplotype Reference Consortium |
| Myocardial infarction | 26343387 | MAF >0.5% | r2 >0.3 or INFO >0.4 | inclusive CAD diagnosis (e.g. myocardial infarction, acute coronary syndrome, chronic stable angina, or coronary stenosis >50%) | logodds | Study-specific covariates and over-dispersion | 1KGP phase 1v3 |
| Large Artery Stroke | 29531354 | MAF ≥1% | INFO ≥ 0.5 | Defined as Trial of Org 10172 in Acute Stroke Treatment | logodds | Study specific , Sex and Age as minimum | 1KGP phase 1v3 or similar |
| Cardioembolic Stroke | 29531354 | MAF ≥1% | INFO ≥ 0.5 | Defined as Trial of Org 10172 in Acute Stroke Treatment | logodds | Study specific , Sex and Age as minimum | 1KGP phase 1v3 or similar |
| Small Vessel Stroke | 29531354 | MAF ≥1% | INFO ≥ 0.5 | Defined as Trial of Org 10172 in Acute Stroke Treatment | logodds | Study specific , Sex and Age as minimum | 1KGP phase 1v3 or similar |

**Supplementary Table 2 Mendelian randomization analysis of Psoriasis and atrial fibrillation.**

| **SNP** | **Chr** | **Position** | **A1** | **A2** | **EAF** | **Psoriasis** | | | **Atrial fibrillation** | | |
| --- | --- | --- | --- | --- | --- | --- | --- | --- | --- | --- | --- |
|  |  |  |  |  |  | **Beta** | **SE** | ***P-*val** | **Beta** | **SE** | ***P-*val** |
| rs10829130 | 10 | 27174346 | A | G | 0.11 | 0.20 | 0.04 | 4.24E-08 | -0.01 | 0.01 | 0.27 |
| rs12713428 | 2 | 61118113 | C | A | 0.25 | 0.17 | 0.03 | 8.11E-11 | 0.01 | 0.009 | 0.12 |
| rs13210419 | 6 | 31266977 | A | G | 0.06 | 1.12 | 0.05 | 1.10E-105 | 0.04 | 0.01 | 0.006 |
| rs138009430 | 16 | 27302897 | A | C | 0.08 | 0.25 | 0.04 | 1.94E-09 | -0.03 | 0.06 | 0.61 |
| rs17728338 | 5 | 150478318 | A | G | 0.07 | 0.31 | 0.04 | 1.76E-12 | 0.03 | 0.02 | 0.03 |
| rs2021511 | 16 | 11344903 | T | C | 0.27 | -0.14 | 0.03 | 4.75E-08 | -0.02 | 0.008 | 0.05 |
| rs60600003 | 7 | 37382465 | G | T | 0.10 | 0.21 | 0.04 | 1.03E-08 | 0.01 | 0.01 | 0.69 |
| rs674451 | 6 | 138216788 | C | T | 0.34 | 0.13 | 0.02 | 2.82E-08 | 0.01 | 0.008 | 0.21 |
| rs9481169 | 6 | 111929862 | T | G | 0.08 | 0.25 | 0.04 | 2.47E-09 | 0.01 | 0.01 | 0.44 |

Abbreviation: SNP, single nucleotide polymorphism; Chr, chromosome; EAF, effect allele frequency; SE, standard error;

Supplementary Table 3 Mendelian randomization analysis of Psoriasis and Heart failure.

| **SNP** | **Chr** | **Position** | **A1** | **A2** | **EAF** | **Psoriasis** | | | **Heart failure** | | |
| --- | --- | --- | --- | --- | --- | --- | --- | --- | --- | --- | --- |
|  |  |  |  |  |  | **Beta** | **SE** | ***P-*val** | **Beta** | **SE** | ***P-*val** |
| rs10829130 | 10 | 27174346 | A | G | 0.11 | 0.20 | 0.04 | 4.24E-08 | -6.00E-04 | 0.012 | 0.96 |
| rs12713428 | 2 | 61118113 | C | A | 0.25 | 0.17 | 0.03 | 8.11E-11 | 0.006 | 0.009 | 0.51 |
| rs13210419 | 6 | 31266977 | A | G | 0.06 | 1.12 | 0.05 | 1.10E-105 | 0.034 | 0.015 | 0.02 |
| rs17728338 | 5 | 150478318 | A | G | 0.07 | 0.31 | 0.04 | 1.76E-12 | 0.001 | 0.017 | 0.95 |
| rs2021511 | 16 | 11344903 | T | C | 0.27 | -0.14 | 0.03 | 4.75E-08 | -0.004 | 0.009 | 0.63 |
| rs60600003 | 7 | 37382465 | G | T | 0.10 | 0.21 | 0.04 | 1.03E-08 | 0.025 | 0.013 | 0.06 |
| rs674451 | 6 | 138216788 | C | T | 0.34 | 0.13 | 0.02 | 2.82E-08 | 0.016 | 0.008 | 0.05 |
| rs9481169 | 6 | 111929862 | T | G | 0.08 | 0.25 | 0.04 | 2.47E-09 | 0.009 | 0.014 | 0.54 |

Abbreviation: SNP, single nucleotide polymorphism; Chr, chromosome; EAF, effect allele frequency; SE, standard error;

Supplementary Table 4 Mendelian randomization analysis of Psoriasis and Myocardial infarction.

| **SNP** | **Chr** | **Position** | **A1** | **A2** | **EAF** | **Psoriasis** | | | **Myocardial infarction** | | |
| --- | --- | --- | --- | --- | --- | --- | --- | --- | --- | --- | --- |
|  |  |  |  |  |  | **Beta** | **SE** | ***P-*val** | **Beta** | **SE** | ***P-*val** |
| rs10829130 | 10 | 27174346 | A | G | 0.11 | 0.20 | 0.04 | 4.24E-08 | 0.01 | 0.01 | 0.48 |
| rs12188300 | 5 | 158829527 | T | A | 0.06 | 0.43 | 0.05 | 2.24E-18 | 0.02 | 0.02 | 0.49 |
| rs12713428 | 2 | 61118113 | C | A | 0.25 | 0.17 | 0.03 | 8.11E-11 | 0.01 | 0.01 | 0.33 |
| rs17728338 | 5 | 150478318 | A | G | 0.07 | 0.31 | 0.04 | 1.76E-12 | 0.04 | 0.02 | 0.05 |
| rs28998802 | 17 | 26124908 | A | G | 0.19 | 0.17 | 0.03 | 7.41E-09 | 0.02 | 0.02 | 0.35 |
| rs60600003 | 7 | 37382465 | G | T | 0.10 | 0.21 | 0.04 | 1.03E-08 | 0.02 | 0.02 | 0.34 |
| rs674451 | 6 | 138216788 | C | T | 0.34 | 0.13 | 0.02 | 2.82E-08 | 0.00 | 0.01 | 0.67 |
| rs9481169 | 6 | 111929862 | T | G | 0.08 | 0.25 | 0.04 | 2.47E-09 | 0.01 | 0.02 | 0.65 |

Abbreviation: SNP, single nucleotide polymorphism; Chr, chromosome; EAF, effect allele frequency; SE, standard error;

Supplementary Table 5 Mendelian randomization analysis of Psoriasis and Valvular heart disease.

| **SNP** | **Chr** | **Position** | **A1** | **A2** | **EAF** | **Psoriasis** | | | **Valvular heart disease** | | |
| --- | --- | --- | --- | --- | --- | --- | --- | --- | --- | --- | --- |
|  |  |  |  |  |  | **Beta** | **SE** | ***P-*val** | **Beta** | **SE** | ***P-*val** |
| rs12188300 | 5 | 158829527 | T | A | 0.06 | 0.43 | 0.05 | 2.24E-18 | 6.55E-04 | 2.67E-04 | 0.01 |
| rs12713428 | 2 | 61118113 | C | A | 0.25 | 0.17 | 0.03 | 8.11E-11 | 2.16E-05 | 1.87E-04 | 0.91 |
| rs1611309 | 6 | 29902063 | T | C | 0.63 | 0.24 | 0.03 | 1.60E-19 | 8.17E-05 | 1.84E-04 | 0.66 |
| rs2021511 | 16 | 11344903 | T | C | 0.27 | -0.14 | 0.03 | 4.75E-08 | -2.58E-04 | 1.75E-04 | 0.14 |
| rs28998802 | 17 | 26124908 | A | G | 0.19 | 0.17 | 0.03 | 7.41E-09 | 1.25E-04 | 2.28E-04 | 0.58 |
| rs4713605 | 6 | 32985992 | A | T | 0.33 | 0.15 | 0.02 | 2.35E-10 | 1.07E-04 | 1.62E-04 | 0.51 |
| rs60600003 | 7 | 37382465 | G | T | 0.10 | 0.21 | 0.04 | 1.03E-08 | 3.37E-04 | 2.61E-04 | 0.20 |
| rs674451 | 6 | 138216788 | C | T | 0.34 | 0.13 | 0.02 | 2.82E-08 | 1.49E-04 | 1.67E-04 | 0.37 |
| rs9481169 | 6 | 111929862 | T | G | 0.08 | 0.25 | 0.04 | 2.47E-09 | 2.78E-04 | 2.83E-04 | 0.33 |

Abbreviation: SNP, single nucleotide polymorphism; Chr, chromosome; EAF, effect allele frequency; SE, standard error;

Supplementary Table 6 Mendelian randomization analysis of Psoriasis and Large Artery Stroke.

| **SNP** | **Chr** | **Position** | **A1** | **A2** | **EAF** | **Psoriasis** | | | **Large Artery Stroke** | | |
| --- | --- | --- | --- | --- | --- | --- | --- | --- | --- | --- | --- |
|  |  |  |  |  |  | **Beta** | **SE** | ***P-*val** | **Beta** | **SE** | ***P-*val** |
| rs10829130 | 10 | 27174346 | A | G | 0.11 | 0.20 | 0.04 | 4.24E-08 | 0.005 | 0.03 | 0.87 |
| rs12188300 | 5 | 158829527 | T | A | 0.06 | 0.43 | 0.05 | 2.24E-18 | 0.01 | 0.05 | 0.78 |
| rs12713428 | 2 | 61118113 | C | A | 0.25 | 0.17 | 0.03 | 8.11E-11 | 0.02 | 0.03 | 0.41 |
| rs13210419 | 6 | 31266977 | A | G | 0.06 | 1.12 | 0.05 | 1.10E-105 | 0.15 | 0.05 | 0.007 |
| rs138009430 | 16 | 27302897 | A | C | 0.08 | 0.25 | 0.04 | 1.94E-09 | 0.02 | 0.10 | 0.88 |
| rs17728338 | 5 | 150478318 | A | G | 0.07 | 0.31 | 0.04 | 1.76E-12 | 0.04 | 0.04 | 0.35 |
| rs2021511 | 16 | 11344903 | T | C | 0.27 | -0.14 | 0.03 | 4.75E-08 | -0.06 | 0.02 | 0.02 |
| rs28752856 | 6 | 31298418 | G | C | 0.11 | 0.83 | 0.04 | 5.90E-100 | 0.05 | 0.04 | 0.22 |
| rs28998802 | 17 | 26124908 | A | G | 0.19 | 0.17 | 0.03 | 7.41E-09 | 0.06 | 0.03 | 0.08 |
| rs60600003 | 7 | 37382465 | G | T | 0.10 | 0.21 | 0.04 | 1.03E-08 | 0.12 | 0.04 | 0.002 |
| rs674451 | 6 | 138216788 | C | T | 0.34 | 0.13 | 0.02 | 2.82E-08 | 0.01 | 0.02 | 0.65 |
| rs9481169 | 6 | 111929862 | T | G | 0.08 | 0.25 | 0.04 | 2.47E-09 | 0.01 | 0.04 | 0.68 |

Abbreviation: SNP, single nucleotide polymorphism; Chr, chromosome; EAF, effect allele frequency; SE, standard error;

Supplementary Table 7 Mendelian randomization analysis of Psoriasis and Cardioembolic Stroke.

| **SNP** | **Chr** | **Position** | **A1** | **A2** | **EAF** | **Psoriasis** | | | **Cardioembolic Stroke** | | |
| --- | --- | --- | --- | --- | --- | --- | --- | --- | --- | --- | --- |
|  |  |  |  |  |  | **Beta** | **SE** | ***P-*val** | **Beta** | **SE** | ***P-*val** |
| rs10829130 | 10 | 27174346 | A | G | 0.11 | 0.20 | 0.04 | 4.24E-08 | -0.007 | 0.03 | 0.79 |
| rs12188300 | 5 | 158829527 | T | A | 0.06 | 0.43 | 0.05 | 2.24E-18 | 6.00E-04 | 0.04 | 0.99 |
| rs12713428 | 2 | 61118113 | C | A | 0.25 | 0.17 | 0.03 | 8.11E-11 | 0.028 | 0.02 | 0.18 |
| rs13210419 | 6 | 31266977 | A | G | 0.06 | 1.12 | 0.05 | 1.10E-105 | -0.010 | 0.05 | 0.82 |
| rs17728338 | 5 | 150478318 | A | G | 0.07 | 0.31 | 0.04 | 1.76E-12 | 0.056 | 0.04 | 0.12 |
| rs2021511 | 16 | 11344903 | T | C | 0.27 | -0.14 | 0.03 | 4.75E-08 | 0.003 | 0.02 | 0.89 |
| rs28752856 | 6 | 31298418 | G | C | 0.11 | 0.83 | 0.04 | 5.90E-100 | 0.021 | 0.03 | 0.52 |
| rs28998802 | 17 | 26124908 | A | G | 0.19 | 0.17 | 0.03 | 7.41E-09 | -0.023 | 0.03 | 0.42 |
| rs60600003 | 7 | 37382465 | G | T | 0.10 | 0.21 | 0.04 | 1.03E-08 | 0.008 | 0.03 | 0.80 |
| rs674451 | 6 | 138216788 | C | T | 0.34 | 0.13 | 0.02 | 2.82E-08 | 0.029 | 0.02 | 0.14 |
| rs9481169 | 6 | 111929862 | T | G | 0.11 | 0.25 | 0.04 | 2.47E-09 | 0.026 | 0.03 | 0.39 |
| rs138009430 | 16 | 27302897 | A | C | 0.08 | 0.25 | 0.04 | 1.94E-09 | 0.02 | 0.10 | 0.88 |

Abbreviation: SNP, single nucleotide polymorphism; Chr, chromosome; EAF, effect allele frequency; SE, standard error;

Supplementary Table 8 Mendelian randomization analysis of Psoriasis and Small Vessel Stroke.

| **SNP** | **Chr** | **Position** | **A1** | **A2** | **EAF** | **Psoriasis** | | | **Small Vessel Stroke** | | |
| --- | --- | --- | --- | --- | --- | --- | --- | --- | --- | --- | --- |
|  |  |  |  |  |  | **Beta** | **SE** | ***P-*val** | **Beta** | **SE** | ***P-*val** |
| rs10829130 | 10 | 27174346 | A | G | 0.11 | 0.20 | 0.04 | 4.24E-08 | -0.020 | 0.04 | 0.57 |
| rs12188300 | 5 | 158829527 | T | A | 0.06 | 0.43 | 0.05 | 2.24E-18 | -0.003 | 0.04 | 0.95 |
| rs12713428 | 2 | 61118113 | C | A | 0.25 | 0.17 | 0.03 | 8.11E-11 | -0.008 | 0.03 | 0.77 |
| rs13210419 | 6 | 31266977 | A | G | 0.06 | 1.12 | 0.05 | 1.10E-105 | -0.004 | 0.06 | 0.94 |
| rs17728338 | 5 | 150478318 | A | G | 0.07 | 0.31 | 0.04 | 1.76E-12 | 0.037 | 0.05 | 0.48 |
| rs2021511 | 16 | 11344903 | T | C | 0.27 | -0.14 | 0.03 | 4.75E-08 | -0.033 | 0.03 | 0.21 |
| rs28752856 | 6 | 31298418 | G | C | 0.11 | 0.83 | 0.04 | 5.90E-100 | 0.045 | 0.05 | 0.35 |
| rs28998802 | 17 | 26124908 | A | G | 0.19 | 0.17 | 0.03 | 7.41E-09 | -0.047 | 0.03 | 0.18 |
| rs4713605 | 6 | 32985992 | A | T | 0.33 | 0.15 | 0.02 | 2.35E-10 | -0.015 | 0.03 | 0.61 |
| rs60600003 | 7 | 37382465 | G | T | 0.10 | 0.21 | 0.04 | 1.03E-08 | -0.014 | 0.04 | 0.72 |
| rs674451 | 6 | 138216788 | C | T | 0.34 | 0.13 | 0.02 | 2.82E-08 | -0.012 | 0.03 | 0.64 |
| rs9481169 | 6 | 111929862 | T | G | 0.08 | 0.25 | 0.04 | 2.47E-09 | -0.012 | 0.04 | 0.77 |

Abbreviation: SNP, single nucleotide polymorphism; Chr, chromosome; EAF, effect allele frequency; SE, standard error;

## Supplementary Figures


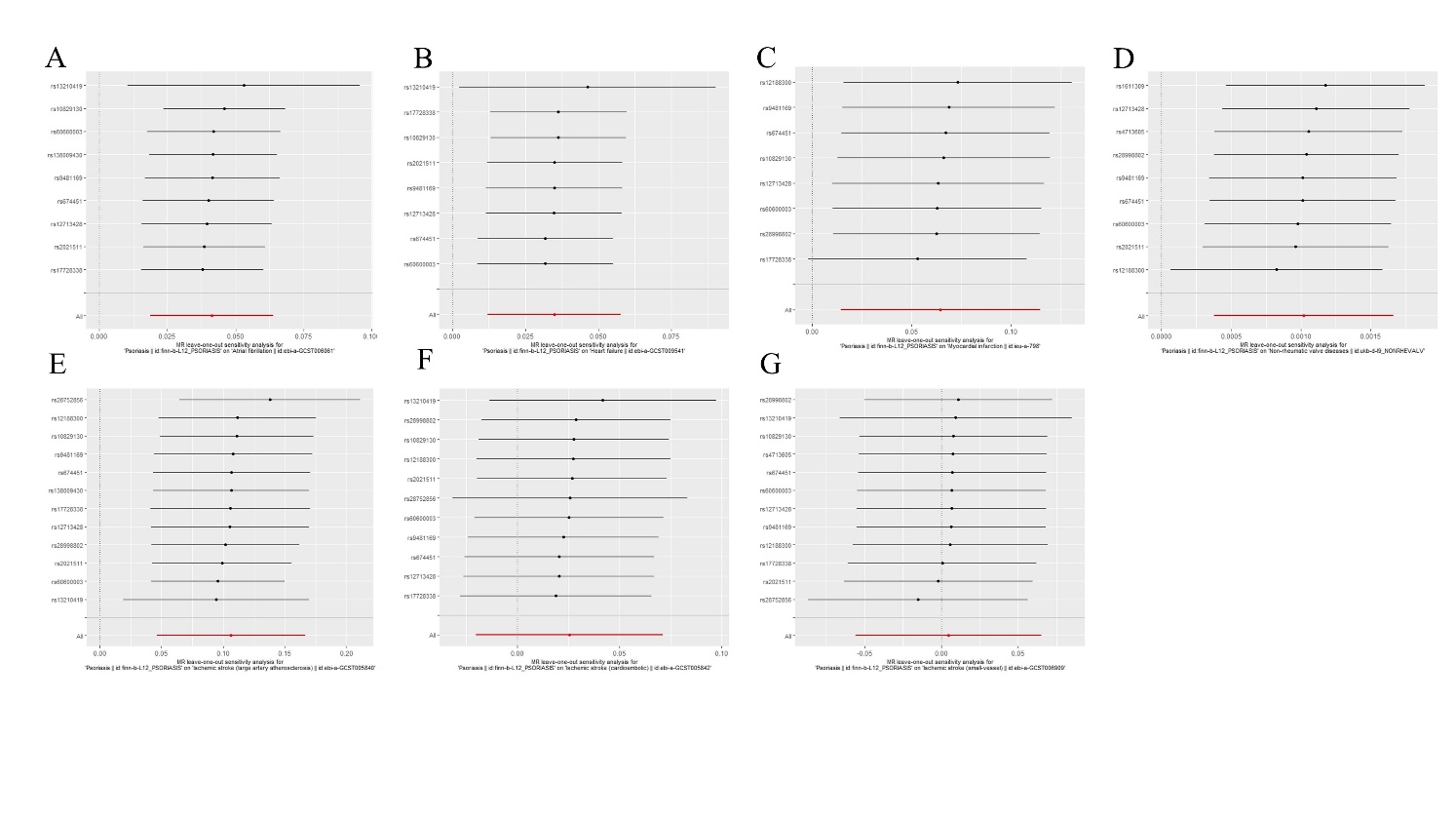


Supplementary Figure 1 Leave-one-out sensitivity analysis: (A) Psoriasis and Atrial fibrillation; (B) Heart failure; (C) Psoriasis and Myocardial infarction; (D) Psoriasis and Myocardial infarction; (E) Psoriasis and Large Artery Stroke; (F) Psoriasis and Cardioembolic Stroke; (G) Psoriasis and Small Vessel Stroke.


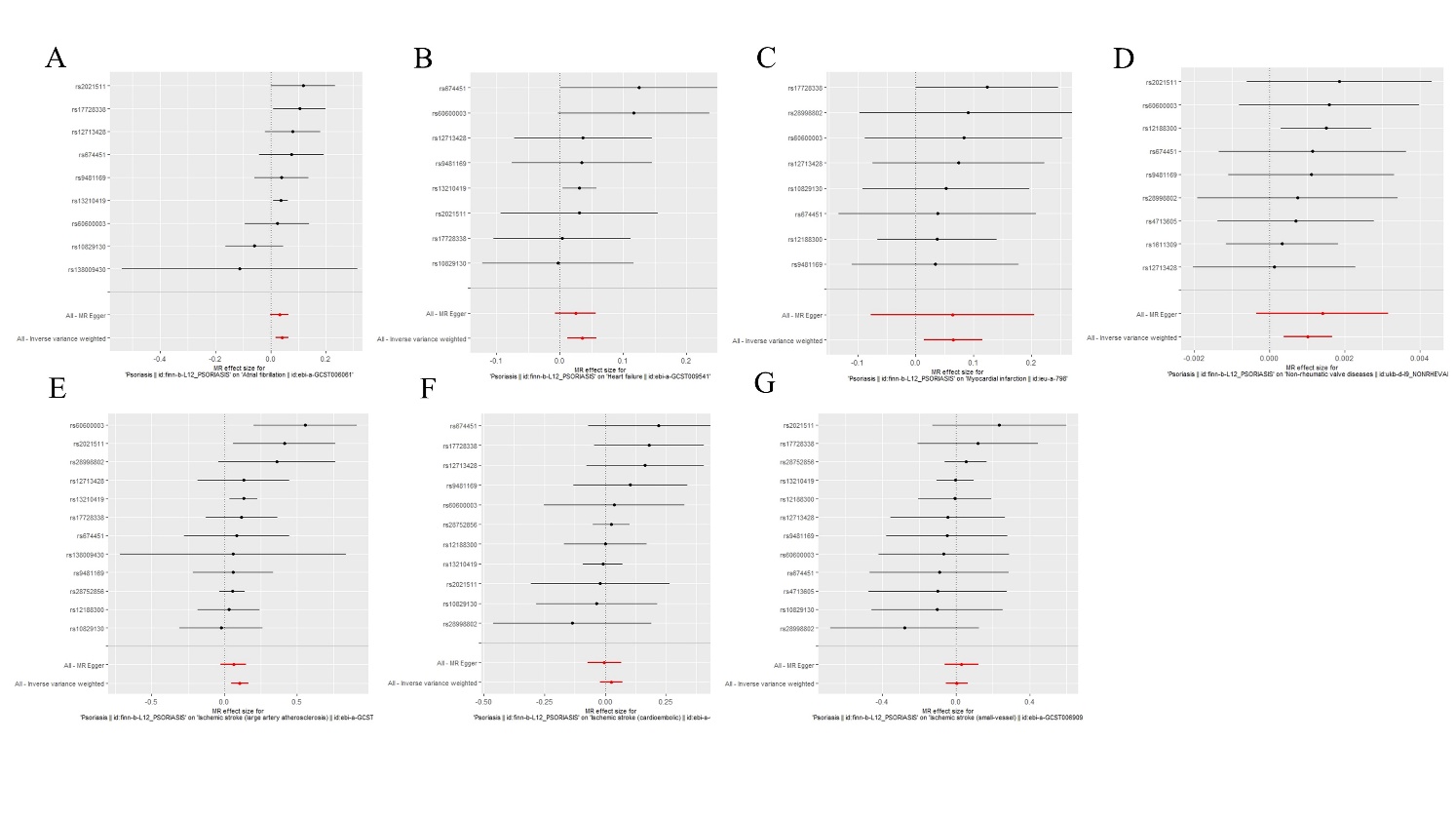


Supplementary Figure 2 Forest plot: (A) Psoriasis and Atrial fibrillation; (B) Heart failure; (C) Psoriasis and Myocardial infarction; (D) Psoriasis and Myocardial infarction; (E) Psoriasis and Large Artery Stroke; (F) Psoriasis and Cardioembolic Stroke; (G) Psoriasis and Small Vessel Stroke.


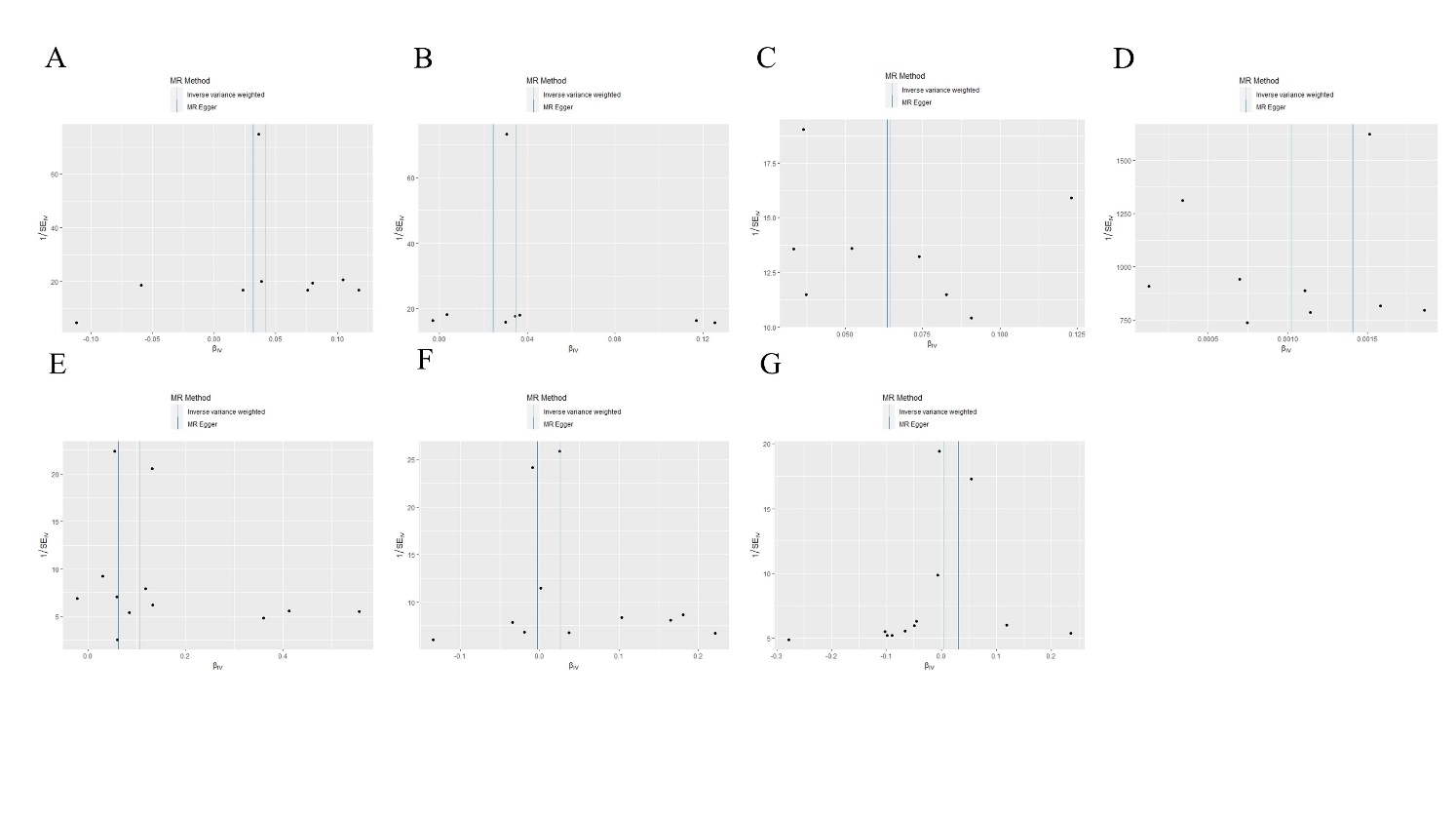


Supplementary Figure 3 Funnel plot: (A) Psoriasis and Atrial fibrillation; (B) Heart failure; (C) Psoriasis and Myocardial infarction; (D) Psoriasis and Myocardial infarction; (E) Psoriasis and Large Artery Stroke; (F) Psoriasis and Cardioembolic Stroke; (G) Psoriasis and Small Vessel Stroke.
